# Green tea extract and its major constituent epigallocatechin-3-gallate inhibit growth and halitosis-related properties of *Solobacterium moorei*

**DOI:** 10.1186/s12906-015-0557-z

**Published:** 2015-03-10

**Authors:** Marie-Pierre Morin, Telma Blanca Lombardo Bedran, Jade Fournier-Larente, Bruno Haas, Jabrane Azelmat, Daniel Grenier

**Affiliations:** Groupe de Recherche en Écologie Buccale, Faculté de Médecine Dentaire, Université Laval, 2420 rue de la Terrasse, G1V 0A6 Quebec City, QC Canada; Department of Oral Diagnosis and Surgery, Araraquara Dental School, State University of São Paulo, São Paulo, Brazil

**Keywords:** Green tea, EGCG, Halitosis, *Solobacterium moorei*

## Abstract

**Background:**

*Solobacterium moorei* is a volatile sulfide compound (VSC)-producing Gram-positive anaerobic bacterium that has been associated with halitosis. The aim of this study was to investigate the effects of green tea extract and its major constituent epigallocatechin-3-gallate (EGCG) on growth and several halitosis-related properties of *S. moorei*.

**Methods:**

A microplate dilution assay was used to determine the antibacterial activity of green tea extract and EGCG against *S. moorei*. Their effects on bacterial cell membrane integrity were investigated by transmission electron microscopy and a fluorescence-based permeability assay. Biofilm formation was quantified by crystal violet staining. Adhesion of FITC-labeled *S. moorei* to oral epithelial cells was monitored by fluorometry. The modulation of β-galactosidase gene expression in *S. moorei* was evaluated by quantitative RT-PCR.

**Results:**

The green tea extract as well as EGCG inhibited the growth of *S. moorei*, with MIC values of 500 and 250 μg/ml, respectively. Transmission electron microscopy analysis and a permeabilization assay brought evidence that the bacterial cell membrane was the target of green tea polyphenols. Regarding the effects of green tea polyphenols on the *S. moorei* colonization properties, it was found that biofilm formation on EGCG-treated surfaces was significantly affected, and that green tea extract and EGCG can cause the eradication of pre-formed *S. moorei* biofilms. Moreover, both the green tea extract and EGCG were found to reduce the adherence of *S. moorei* to oral epithelial cells. The β-galactosidase activity of *S. moorei*, which plays a key role in VSC production, was dose-dependently inhibited by green tea polyphenols. In addition, EGCG at ½ MIC significantly decreased the β-galactosidase gene expression.

**Conclusion:**

Our study brought evidence to support that green tea polyphenols possess a number of properties that may contribute to reduce *S. moorei*-related halitosis. Therefore, these natural compounds may be of interest to be used to supplement oral healthcare products.

## Background

Tea, an aqueous aromatic infusion of dried leaves of the plant *Camellia sinensis*, is the most widely consumed beverage in the world after water. More specifically, green tea is made of non-fermented leaves and has a high catechin content (flavan-3-ols), including epigallocatechin-3-gallate (EGCG) that represents about 59% of total catechins [1]. There is an emerging body of evidence supporting that green tea polyphenols may contribute to reducing the risk and/or severity of many systemic conditions and diseases, such as diabetes, cardiovascular disease and cancer [1-3]. The beneficial properties of green tea polyphenols have been mostly associated to their anti-inflammatory, anti-mutagenic, and anti-oxidative properties [1-3].

Epidemiological and clinical studies have provided some evidence that green tea consumption may have potential oral health benefits thus resulting in a decreased incidence/severity of dental caries and periodontal diseases, the two most common oral infections [4-8]. On the one hand, the ability of green tea polyphenols to inhibit *Streptococcus mutans* growth, biofilm formation, and acid production may contribute to its beneficial effects for dental caries [9-11]. On the other hand, the positive impact of green tea polyphenols for periodontal diseases may be associated to their ability to inhibit growth, adherence, and protease activity of *Porphyromonas gingivalis* [12,13], a major pathogen causing chronic periodontitis. Moreover, it has been shown that green tea polyphenols can exert anti-inflammatory properties by decreasing the secretion of interleukin-6, interleukin-8, and C-C motif chemokine ligand 5 (CCL5) by *P. gingivalis*-stimulated gingival epithelial cells [13]. Recently, our laboratory reported that green tea extract and its major constituent EGCG induce the secretion of β-defensins, a family of antimicrobial peptides, by gingival epithelial cells [14]. This property of green tea polyphenols to increase epithelial β-defensin secretion may have a positive impact by strengthening the epithelial antimicrobial barrier.

Halitosis (oral malodor) that affects a large proportion of the population is a condition in which a person suffers from bad breath. Bad breath originating from the oral cavity is due to volatile sulfide compounds (VSCs) generated through bacterial metabolism [15,16]. The dorso-posterior region of the tongue is a major source of oral malodor [17]. More particularly, proteolytic bacteria found in tongue coating degrade proteins leading to the production of cysteine and methionine which are further processed into hydrogen sulfide and methylmercaptan by specific bacterial species [15,16]. The basic treatment for oral malodor includes mechanical removal of the tongue coating with toothbrushes or tongue scrapers [18]. In addition, the use of oral care products supplemented with chemicals effective in preventing bacterial growth (chlorhexidine, cetylpyridinium chloride, triclosan, essential oils) or neutralizing VSCs (chlorine dioxide, zinc salts) can improve the condition significantly [18]. Few studies have also brought evidence that green tea polyphenols may be effective in reducing halitosis [19,20]. The efficacy of green tea polyphenols for halitosis has been related to their ability to modify VSCs that results in a deodorant activity [19,21].

The Gram-positive anaerobic bacterium *Solobacterium moorei* has been specifically associated with oral malodor since it has been reported to be present in subjects with halitosis but not in control subjects [22-24]. As a matter of fact, Haraszthy et al. [22] detected *S. moorei* in 100% of the 21 subjects with halitosis compared to only 14% of the control subjects. Recently, Vancauwenberghe et al. [25] reported a significant correlation between *S. moorei*, tongue coating and total VSCs. In a previous study, we showed that *S. moorei* can be a major source of malodorous compounds by producing VSCs from mucin through a process involving the cell-associated β-galactosidase activity of the bacterium and an exogenous source of proteases [26]. In this study, we investigated the effects of a green tea extract and its major constituent EGCG on growth and several halitosis-related properties of *S. moorei*.

## Methods

### Green tea extract and epigallocatechin-3-gallate

The commercial green tea extract (Organic Herb Inc., Changsha, China) used in this study had a polyphenol content ≥ 98%, including 45% EGCG, according to the company’s data sheet. A stock solution was freshly prepared by dissolving 20 mg of powder in one ml of sterile warm distilled water and filtering the solution through a 0.2 μm-pore membrane filter. EGCG (Sigma-Aldrich Canada Ltd, Oakville, ON, Canada), the major catechin in green tea, was also dissolved in sterile distilled water at a concentration of 5 mg/ml and was filter sterilized as above.

### Bacteria and culture conditions

*S. moorei* CH8-20, kindly provided by V. Haraszthy (The State University of New York at Buffalo), was used in this study. This strain was isolated from the dorsal surface of the tongue in a subject with halitosis [22]. Bacteria were routinely grown in Todd-Hewitt Broth (THB) medium (BBL Microbiology Systems, Cockeysville, MD, USA) supplemented with 0.001% hemin, 0.0001% vitamin K, 0.5% Tween-80, 0.2% yeast extract, and 1% glucose. Incubation was carried out at 37°C under anaerobic conditions (N_2_:H_2_:CO_2_/75:10:15).

### Determination of minimal inhibitory concentrations and minimal bactericidal concentrations

Minimal inhibitory concentrations (MICs) and minimal bactericidal concentrations (MBCs) were determined using a microbroth dilution assay as described in a previous study [27]. Penicillin G was used as reference compound. MIC values (μg/ml) of compounds were determined as the lowest concentration at which no growth occurred. To determine MBC values (μg/ml), aliquots (5 μl) of each well showing no visible growth were spread on blood-supplemented THB agar plates, which were incubated for 3 days at 37°C. MBCs of compounds were determined as the lowest concentration at which no colony formation occurred. The MIC and MBC values were determined in three independent experiments.

### Transmission electron microscopy analysis of bacterial cells

*S. moorei* was grown as above, harvested by centrifugation, and washed once in 50 mM phosphate-buffered saline pH 7.2 (PBS). Cells were suspended in PBS at an OD_660_ of 0.5 and incubated in the presence of either green tea extract (500 μg/ml) or EGCG (250 μg/ml) at room temperature for 4 h. Thereafter, bacteria were fixed for 2 h at room temperature in 0.1 M cacodylate buffer (pH 7) containing 5% glutaraldehyde and 0.15% ruthenium red. Cells were then reacted with polycationic ferritin (1 mg/ml) and processed as described by Vanrobaeys et al. [28]. Thin sections were examined using a JEOL 1230 transmission electron microscope at an accelerating voltage of 80 kV.

### Cell membrane permeability assay

The effect of green tea extract and EGCG on cell membrane permeability of *S. moorei* was determined using the intracellular dye calcein acetoxymethyl ester (calcein-AM) (Sigma-Aldrich Canada Ltd), as previously described [29]. Briefly, *S. moorei* cells were suspended in PBS at an OD_660_ of 0.1 and one ml was incubated in the presence of 5 μl of 1 mM calcein-AM for 4 h at room temperature. Bacteria were then washed twice and suspended in 2 ml of PBS. Calcein-AM-loaded bacteria were dispensed (100 μl) into wells of a black 96-well microplate, and incubated at room temperature in the presence of either green tea extract (500 μg/ml) or EGCG (250 μg/ml). The release of calcein-AM resulting from cell damages was monitored every 10 min during 160 min using a microplate reader at excitation wavelength of 485 nm and emission wavelength of 530 nm. PBS was used as negative control while heat-treated (80°C/10 min) cells were used as positive control.

### Biofilm formation and desorption

The effect of treating wells of a microplate with either green tea extract or EGCG (1000 to 3.125 μg/ml) for 2 h (room temperature) on biofilm formation by *S. moorei* was assessed. Wells treated with PBS served as control. Following treatment, a 24-h culture of *S. moorei* was diluted in fresh broth medium to obtain an OD_660_ of 0.1. Samples (200 μl) were added to treated wells of a 96-well microplate. After incubation for 48 h at 37°C under anaerobic conditions, spent media and free-floating bacteria were removed by aspiration using a 26 g needle. The wells were washed once with PBS and the *S. moorei* biofilms were stained with 0.05% crystal violet (100 μl) for 15 min. The wells were washed four times with PBS to remove unbound crystal violet dye and dried for 2 h at 37°C. After adding 100 μl of 95% (v/v) ethanol to each well, the plate was shaken for 10 min to release the stain from the biofilms and the absorbance at 550 nm (A_550_) was recorded. In addition, the capacity of green tea extract and EGCG to promote biofilm desorption was investigated. Briefly, 48-h pre-formed *S. moorei* biofilms in microplate were treated for 2 h under anaerobiosis with either green tea extract or EGCG (1000 to 31.25 μg/ml). Following these treatments, the biofilms were washed twice with PBS and stained with crystal violet as above. Biofilms treated with PBS were used as control.

### Adherence assay to oral epithelial cells

*S. moorei* cells cultivated as above were labeled with fluorescein isothyocyanate (FITC) as previously reported [30]. The immortalized human oral epithelial cell line OBA-9 used in this study, kindly provided by Dr. Marcia Mayer (Departamento de Microbiologia, Institute of Biomedical Sciences, Universidade de São Paulo, São Paulo, Brazil), was initially described by Kusumoto *et al.* [31]. The epithelial cells were cultured (96-well microplate) in Keratinocyte-Serum Free Medium (K-SFM, Life Technologies Inc., Burlington, ON, Canada) containing insulin, epidermal growth factor, and fibroblast growth factor, and supplemented with 100 μg/ml of penicillin G/streptomycin at 37°C in a 5% CO_2_ atmosphere until they reached confluence. The adherence assay of *S. moorei* to epithelial cells in the absence or presence of green tea extract or EGCG (200 to 3.125 μg/ml) was carried out as described in a previous study [13]. After removing unbound bacteria and washing wells, the relative fluorescence units (RUF; excitation wavelength 495 nm; emission wavelength 525 nm) corresponding to the degree of bacterial adherence were determined using a microplate reader.

### Determination of β-galactosidase activity

The effect of green tea extract and EGCG on cell-associated β-galactosidase activity of *S. moorei* was determined using the chromogenic substrate *o*-nitrophenyl-β-d-galactopyranoside (Sigma-Aldrich Canada Ltd). Briefly, cells of *S. moorei* (OD_660_ = 2.0 in PBS) (100 μl) were incubated with 25 μl of substrate (2 mg/ml) and 25 μl of green tea extract or EGCG (500 to 62.5 μg/ml). The reaction mixtures were incubated at 37°C for 2 h and bacteria were removed by centrifugation prior to measure the absorbance at 405 nm (A_405_).

### Determination of β-galactosidase gene expression by quantitative RT-PCR

To investigate the effect of green tea extract and EGCG on β-galactosidase gene expression, *S. moorei* was grown to mid-log phase (OD_660_ = 0.4) and then compounds were added at MIC (500 and 250 μg/ml for green tea extract and EGCG, respectively) and ½ MIC (250 and 125 μg/ml for green tea extract and EGCG, respectively) prior to further incubate at 37°C for 8 h. Bacteria were collected by centrifugation (7000 × *g* for 5 min) and treated with an RNAprotect bacterial reagent (Qiagen Canada Inc., Montreal, QC, Canada). Bacterial cells were then lysed and RNA was isolated and purified using the RNeasy minikit (Qiagen Canada Inc.). The amounts of mRNA were quantified with the Experion™ system (Bio-Rad Laboratories, Mississauga, ON, Canada). The reverse transcription-polymerase chain reaction (RT-PCR) to prepare cDNA was performed using the iScript™ Reverse Transcription Supermix (Bio-Rad Laboratories) according to the manufacturer. Reverse transcription conditions were 5 min at 25°C, 30 min at 42°C, and 5 min at 85°C. Real-time PCR was used for quantification of β-galactosidase mRNA expression. 16S rRNA gene was used as an internal control for data normalization. The primers used for the quantitative RT-PCR were designed in this study and purchased from Life Technologies Inc. The sequences of the forward and reverse primers used were 16SSmo-F: 5′-CTGTAGAGATACAGTAGAGGTTATC-3′ and 16SSmo-R: 5′-ATTGTAGTACGTGTGTAGCC-3′, respectively, for 16S rRNA gene, and 1477Bgal778: 5′-GTATTCTTGATAGGTCTAAATCGTC-3′ and 1477Bgal946R: 5′-CAGTAAATACATACAAACCATAACG-3′, respectively, for β-galactosidase gene. Triplicate reactions were prepared with 10 μl of PCR mixture containing 5 μl of IQ SYBR Green Supermix (Bio-Rad Laboratories), 4 μl of cDNA, 0.4 μl of gene-specific primer, and 0.6 μl of RNase- and DNase-free water. The samples were amplified using a Bio-Rad MyCycler™ thermal cycler (Bio-Rad Laboratories). The amplification conditions were 95°C for 5 min followed by 35 cycles at 95°C for 1 min, 52°C for 1 min and 72°C for 30 s. To validate the specificity of each primer pair, temperature curve analyses were performed.

### Statistical analysis

All assays were performed in triplicate and the means ± standard deviations were calculated. Differences between means were analyzed for statistical significance using the Student’s *t*-test and were considered significant at P < 0.01.

## Results

As reported in Table 1, both green tea extract and EGCG exhibited antibacterial activity towards *S. moorei*. On the one hand, the green tea extract had a MIC of 500 μg/ml while the MBC was 4000 μg/ml. On the other hand, the MIC of EGCG was slightly lower at 250 μg/ml although the MBC was found to be > 4000 μg/ml. As expected, penicillin G used as a reference control showed much lower values of MIC (0.098 μg/ml) and MBC (6.25 μg/ml).

**Table 1:** **Minimal inhibitory concentration (MIC) and minimal bactericidal concentration (MBC) values of green extract, EGCG, and penicillin G for** ***S. moorei***

**Compound**	**MIC (μg/ml)**	**MBC (μg/ml)**
Green tea extract	500	4000
EGCG	250	> 4000
Penicillin G	0.098	6.25

**Figure 1:**
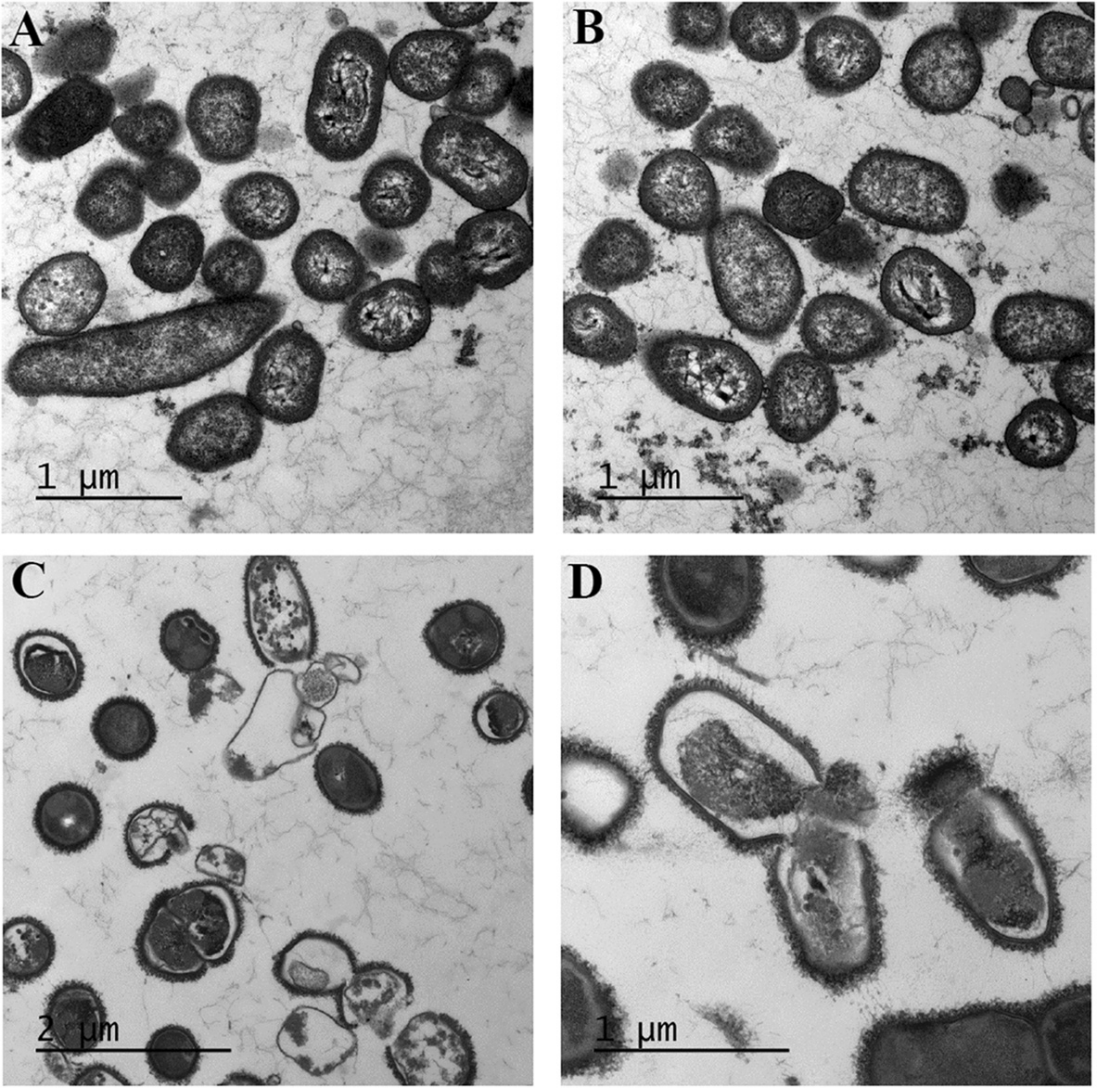
**Transmission electron microscopy analysis of** ***S. moorei*** **.** Panel **A**: Control untreated bacteria. Panel **B**: Bacteria treated (4 h) with green tea extract (500 μg/ml). Panels **C** and **D**: Bacteria treated (4 h) with EGCG (250 μg/ml).

**Figure 2:**
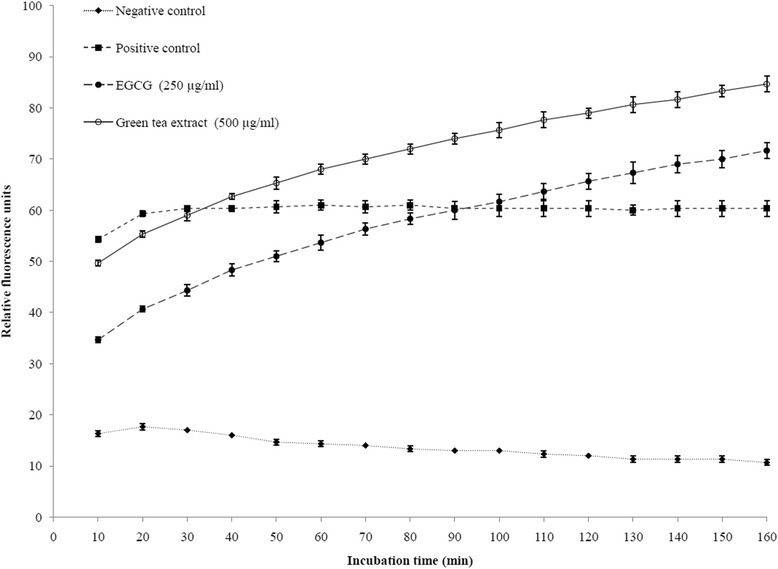
**Time course of green tea extract- and EGCG-induced release of calcein-AM from** ***S. moorei*** **cells.** Cells treated with PBS or heat (80°C/10 min) were used as negative and positive controls, respectively. Data are expressed as means ± standard deviations.

The effect of green tea polyphenols on the cell integrity of *S. moorei* was evaluated by transmission electron microscopy (Figure 1). The ultrastructure of *S. moorei* cells treated with green tea extract at the MIC (500 μg/ml) was not obviously affected compared to control cells (Figure 1A,B). However, in the electron micrographs of EGCG-treated *S. moorei* cells, severe ultrastructural damages were observed, which result in breakdown of the cytoplasmic membrane and cell wall with leakage of the cytoplasmic content (Figure 1C,D). In order to further investigate the effects of green tea polyphenols on the cytoplasmic membrane of *S. moorei*, the fluorescent dye calcein-AM was used. The addition of green tea extract or EGCG, both at MIC, to calcein-AM-loaded *S. moorei* cell suspensions caused a time-dependent release of fluorescence (Figure 2). The release of calcein-AM induced by green tea extract was more significant compared to EGCG.

**Figure 3:**
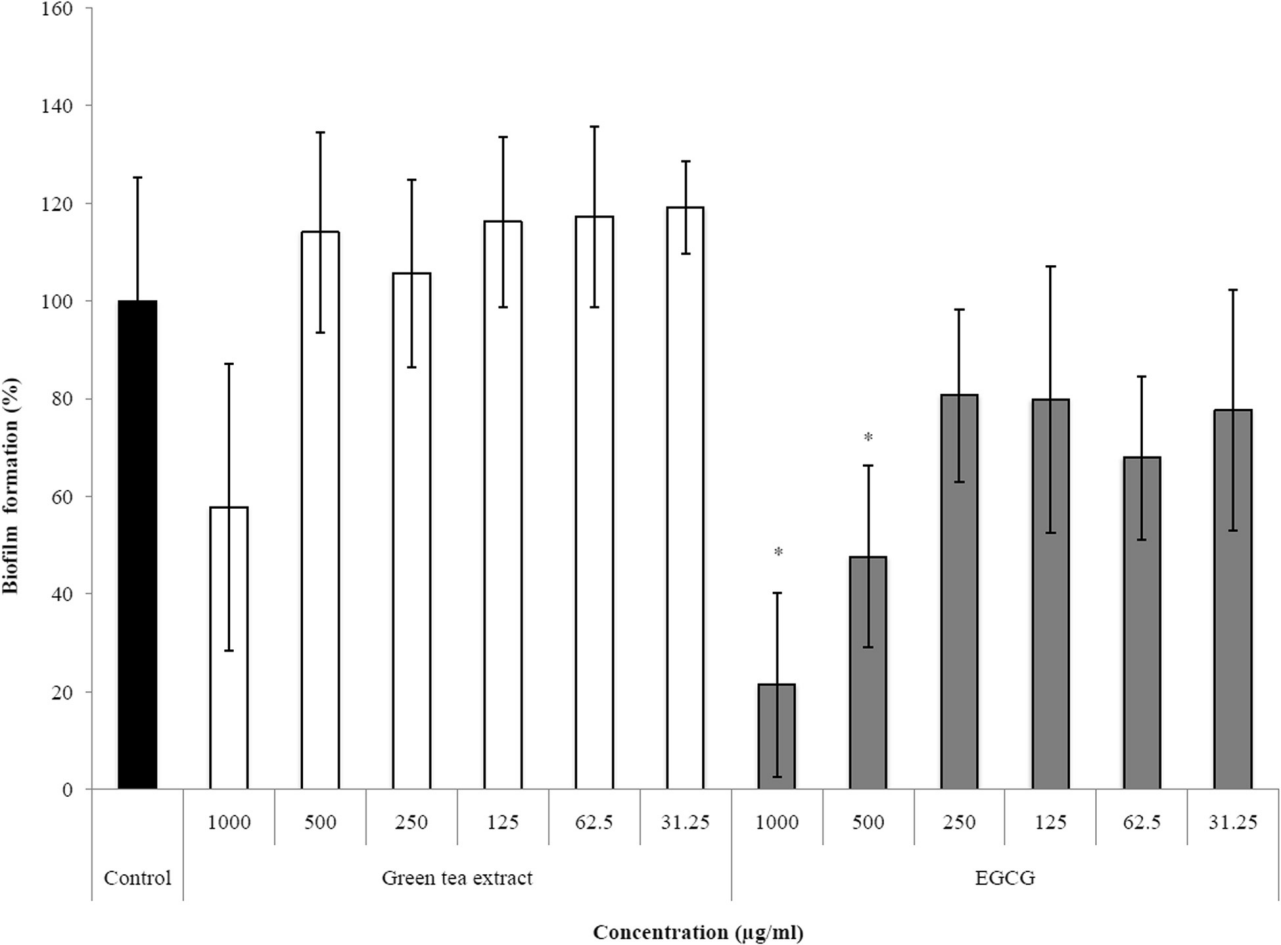
**Effect of green tea extract and EGCG surface pre-treatments on biofilm formation by** ***S. moorei*** **in a microplate assay model.** A relative value of 100% was assigned to control in which wells were pre-treated with PBS. Data are expressed as means ± standard deviations. *, significant inhibition (P < 0.01) compared to control.

When growth of *S. moorei* was carried out in the presence of either green tea extract or EGCG, no specific anti-biofilm effect was observed; the decreased biofilm formation was related to growth inhibition caused by the green tea polyphenols (data not shown). However, as reported in Figure 3, a pre-treatment of the polystyrene surfaces of the microplate with EGCG was found to dose-dependently reduce biofilm formation. Such treatment did not affect bacterial growth. More specifically, pre-treating the polystyrene wells with EGCG at 500 μg/ml reduced *S. moorei* biofilm formation by 52%. Although treatment of wells with the highest concentration of the green tea extract decreased biofilm formation by *S. moorei*, the inhibition was not significant.

**Figure 4:**
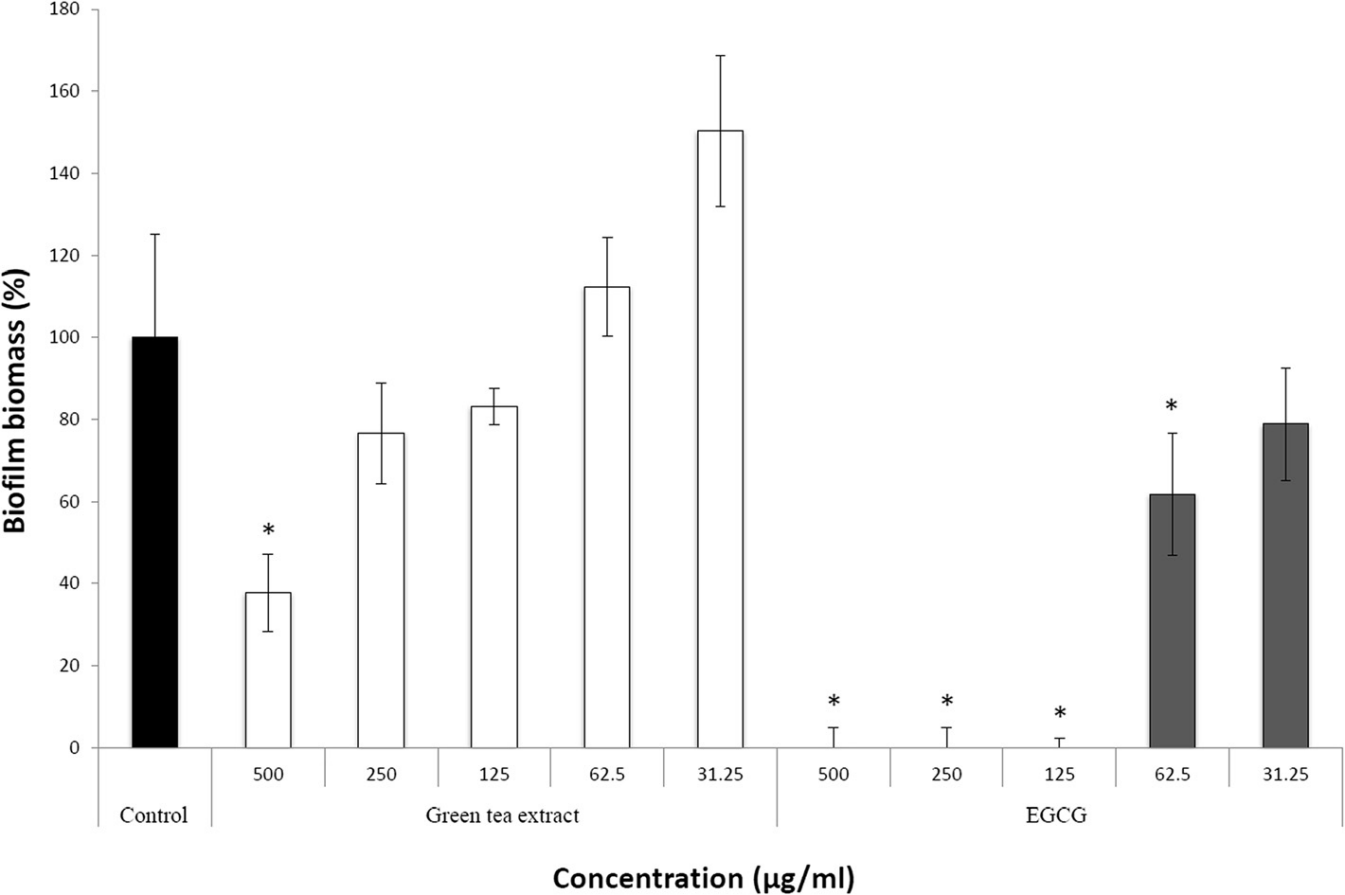
**Effect of green tea extract and EGCG on desorption of a pre-formed** ***S. moorei*** **biofilm in a microplate assay model.** A relative value of 100% was assigned to control in which biofilms were pre-treated with PBS. Data are expressed as means ± standard deviations. *, significant inhibition (P < 0.01) compared to control.

The ability of green tea polyphenols to induce desorption of a pre-formed *S. moorei* biofilm was then investigated. EGCG and to a lesser extent green tea extract were found to dose-dependently desorb biofilms of *S. moorei* following an exposition of 2 h (Figure 4). This effect was found to be more pronounced for EGCG. At 125 μg/ml, EGCG completely eradicated *S. moorei* biofilms, while at the same concentration the green tea extract had no significant effect.

**Figure 5:**
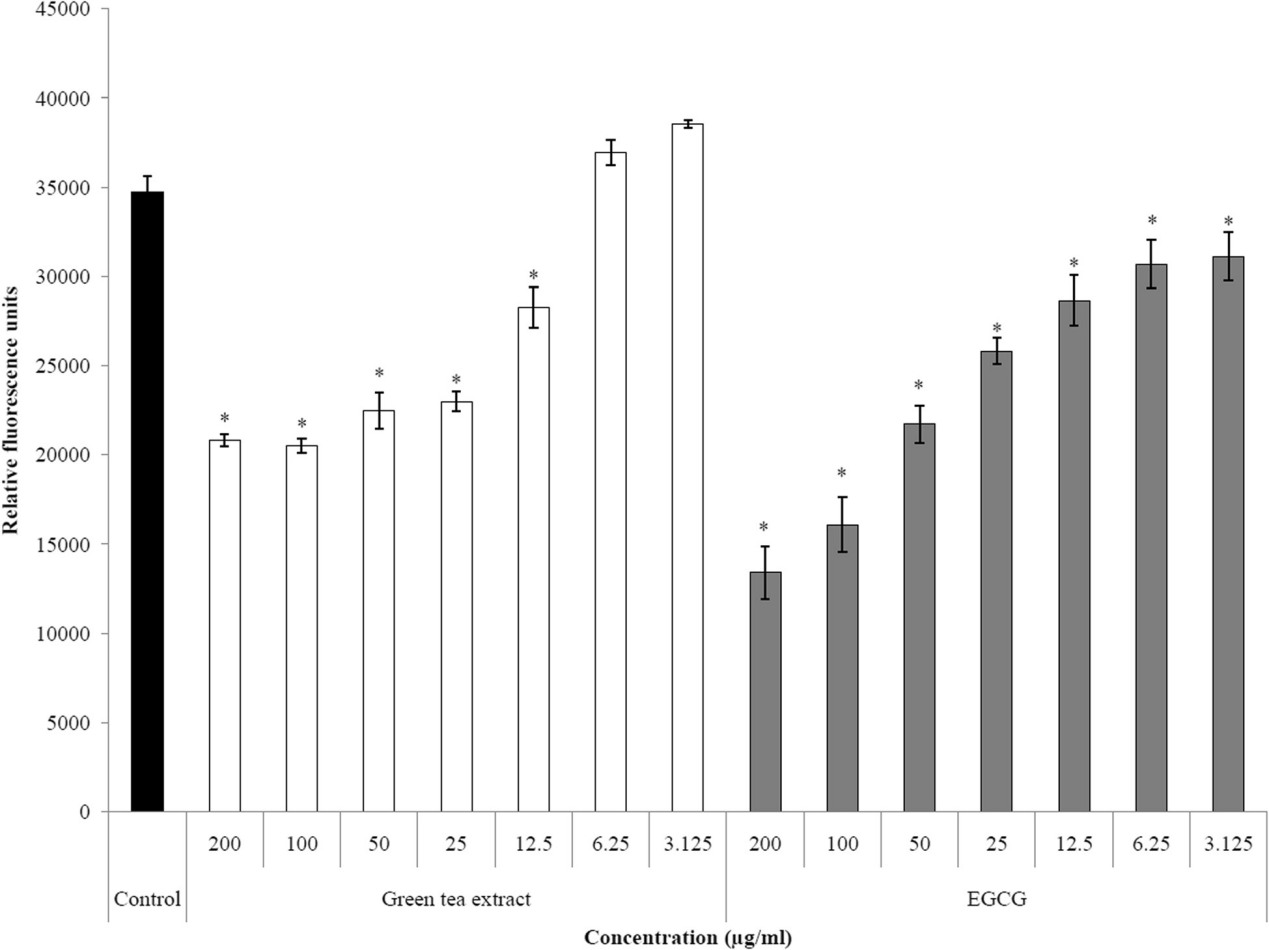
**Effect of green tea extract and EGCG on adherence of** ***S. moorei*** **to oral epithelial cells.** Data are expressed as means ± standard deviations. *, significant inhibition (P < 0.01) compared to control.

Using fluorescein-labelled bacterial cells, *S. moorei* was found to adhere to oral epithelial cells (Figure 5). When the adherence assay was performed in the presence of either green tea extract or EGCG, the adherence of *S. moorei* to oral epithelial cells was dose-dependently inhibited (Figure 5). At the highest concentration tested (200 μg/ml) the inhibition caused by EGCG was more significant.

**Figure 6:**
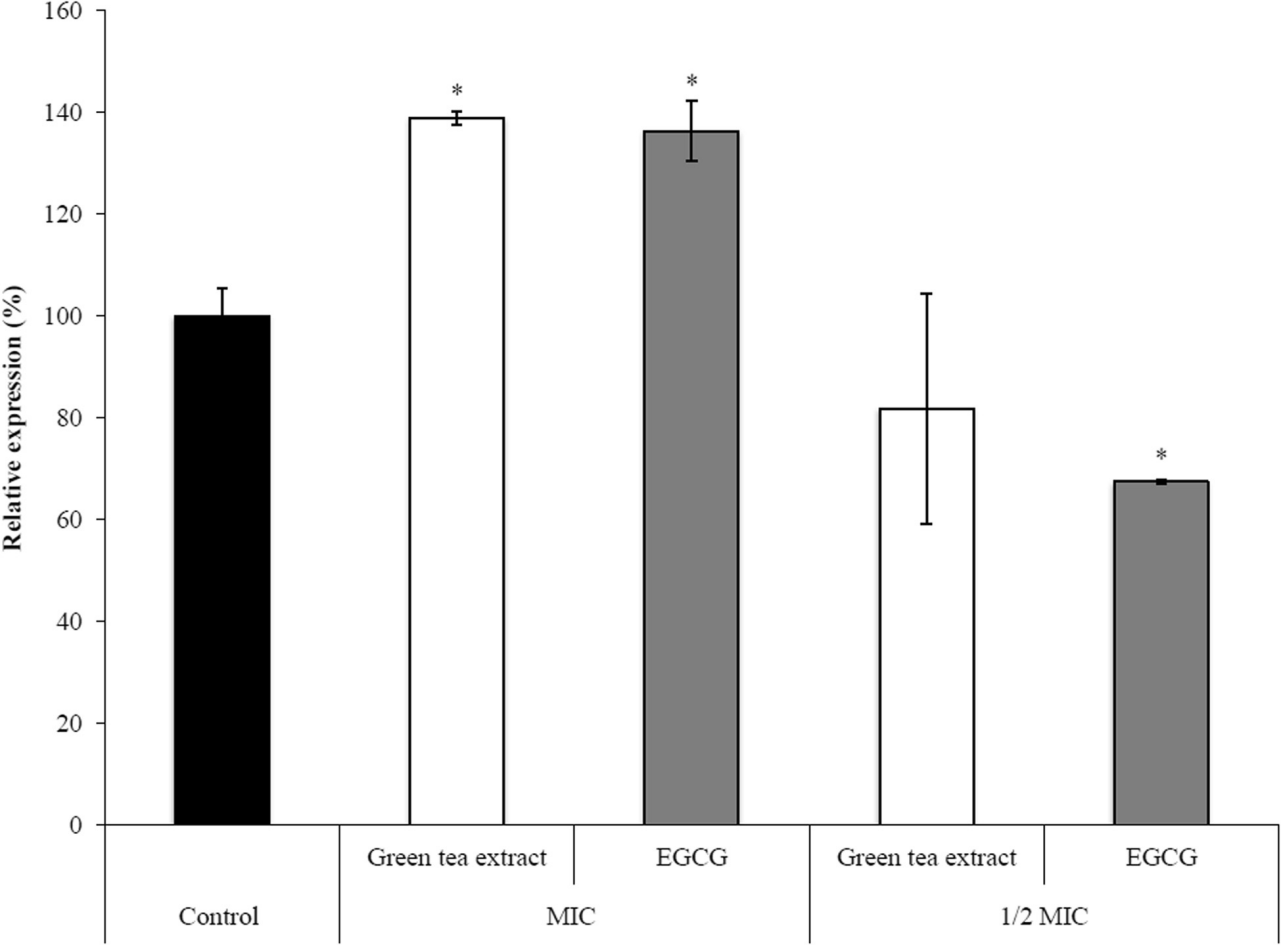
**Effect of green tea extract and EGCG on mRNA expression of β-galactosidase gene in** ***S. moorei*** **.** Bacteria were incubated (8 h) in the presence of compounds at MIC (500 and 250 μg/ml for green tea extract and EGCG, respectively), and ½ MIC (250 and 125 μg/ml for green tea extract and EGCG, respectively). Data are expressed as means ± standard deviations. The expression was normalized to 16S rRNA. *, significantly different (P < 0.01) compared to untreated control.

Given that a previous study [26] showed that the β-galactosidase activity of *S. moorei* plays a key role in VSC production by the bacterium, we tested whether green tea polyphenols can inhibit the activity and gene expression of β-galactosidase. Table 2 reports that both green tea extract and EGCG, at a concentration ≥ 125 μg/ml, caused a significant inhibition of *S. moorei* cell-associated β-galactosidase activity. More specifically, at 250 μg/ml green tea extract and EGCG caused an inhibition of 66.7% and 61.5%, respectively. The effect of green tea polyphenols on β-galactosidase gene expression, as determined by quantitative PCR, is reported in Figure 6. On the one hand, when used at their MIC, both green tea extract and EGCG increased β-galactosidase gene expression. On the other hand, at ½ of their MIC, while green tea extract had no effect, EGCG significantly decreased (33%) the gene expression of β-galactosidase.

**Table 2:** **Effect of green tea extract and EGCG on β-galactosidase activity of** ***S. moorei***

**Compound**		**% inhibition of β-galactosidase activity**
None (control)		0
Green tea extract	500 μg/ml	75.4 ± 9.3*
	250 μg/ml	66.7 ± 11.2*
	125 μg/ml	43.6 ± 13.7*
	62.5 μg/ml	17.3 ± 10.1
EGCG	500 μg/ml	72.1 ± 9.8*
	250 μg/ml	61.5 ± 15.7*
	125 μg/ml	53 ± 9.5*
	62.5 μg/ml	26.4 ± 12.3

*Significantly different at P < 0.01 compared to control.

## Discussion

Halitosis or oral malodor is in most cases caused by the production of VSCs, particularly methyl mercaptan and hydrogen sulfide, by specific bacterial species colonizing the gingival sulcus as well as the dorso-posterior region of the tongue [15]. It is estimated that up to half of the adult population is affected by halitosis, although epidemiologic studies using objective criteria showed that the prevalence may be lower [32-34]. Given its high prevalence in halitosis subjects, the Gram-positive strictly anaerobic bacterium *S. moorei* is considered as a key causative agent of halitosis [22-25]. In addition, it has also been associated with various types of dental infections such as endodontic infections [35,36], and refractory periodontitis [37]. Considering the high incidence of halitosis in the general population, the strong association of *S. moorei* with this condition, and the growing interest of companies for natural plant molecules as efficacious and safe substances for oral care products (mints, gum, mouthwash, etc.), this study examined the effects of green tea extract and EGCG on growth and various halitosis-related properties of *S. moorei*.

Green tea, the non-fermented form of tea, is considered as a functional food since it has positive health effects that extend beyond its nutritional value [8]. The most abundant polyphenol of green tea and the one that has been extensively studied is EGCG [1]. More specifically, Wu and Wei [4] reported that a cup of green tea (2.5 g of green tea leaves/200 ml of water) may contain up to 90 mg of EGCG. Recent studies have suggested that green tea polyphenols may be regarded as molecules of interest for the management of oral diseases, including dental caries and periodontitis [4-13]. In this study, we uncovered new properties associated with green tea polyphenols, including EGCG, that support their potential for controlling halitosis.

We first showed that a green tea extract as well as its main constituent EGCG can efficiently inhibit the growth of *S. moorei*, with MICs of 500 and 250 μg/ml, respectively. In regard to MBCs, a value of 4000 μg/ml was obtained for green tea extract while that of EGCG was >4000 μg/ml. This suggests that green tea extract may contain additional bioactive components, in addition to EGCG, that can exert bactericidal action. These values are in agreement with those previously reported for other oral pathogens, including *P. gingivalis* [12]. Since the cell membrane is a target of many plant polyphenols, we investigated the effect of green tea polyphenols on *S. moorei* cell integrity by transmission electron microscopy and a membrane permeabilization assay. While the green tea extract did not markedly affect the bacterial ultrastructure, EGCG was found to induce major damages. Both green tea extract and EGCG appear to permeabilize the cell membrane resulting in calcein-AM leakage. Previous studies also reported that tea theaflavins and catechins can irreversibly damage the bacterial cytoplasmic membrane [38-40]. In regard to EGCG, it generates hydrogen peroxide in the lipid bilayer of the bacterial cytoplasmic membrane, resulting in leakage of intracellular materials [40]. Additional mechanisms by which green tea polyphenols inhibit the growth of *S. moorei* may be involved. For instance, Navarro-Martinez et al. [41] provided evidence that the antibacterial action of catechins against *Stenotrophomonas maltophila*, a Gram-negative opportunistic pathogen, is due to its ability to inhibit cytoplasmic dihydrofolate reductase. Dihydrofolate reductase reduces dihydrofolic acid to tetrahydrofolic acid, which is required by bacteria to synthetize purine, thymidylate, and nucleic acid precursors, which are very important for cell proliferation and growth.

Biofilm formation by *S. moorei* is likely a key step in halitosis. Although green tea polyphenols did not show any specific anti-biofilm effect on *S. moorei*, we showed that it can affect the biofilm via two mechanisms. First, the formation of biofilm by *S. moorei* on EGCG-treated surfaces was significantly affected. Second, both green tea extract and EGCG can cause the eradication of pre-formed *S. moorei* biofilms. The mechanism involved in biofilm desorption may be related to the ability of green tea polyphenols to alter the cell integrity of *S. moorei*.

Both the green tea extract and EGCG were also found to reduce the adherence of *S. moorei* to oral epithelial cells. This is in agreement with previous data reported by our laboratory showing that tea extracts (green, white, black, and oolong) can prevent the adherence of *P. gingivalis* to epithelial cells [13]. This inhibition may be related to the binding of green tea polyphenols to the bacterial cell surface thus altering the hydrophobicity or hiding adhesins.

In a previous study, we showed that *S. moorei* produces high levels of VSCs when cultivated in the presence of cysteine and that its cell-associated β-galactosidase is essential for VSC production from mucin [26]. This enzyme can deglycosylate salivary glycoproteins, which can be further degraded into peptides and amino acids by host and bacterial proteolytic enzymes prior to be transformed into VSCs. Interestingly, β-galactosidase activity in saliva has been associated with halitosis [42,43]. In this study, we showed that green tea extract and EGCG inhibit β-galactosidase activity of *S. moorei*, a property that may contribute to reduce oral malodor. The ability of green tea polyphenols to attenuate the β-galactosidase gene expression in *S. moorei* was also investigated. It was found that when used at MIC, both the green tea extract and EGCG up-regulate gene expression while at ½ MIC, EGCG significantly decreased the β-galactosidase gene expression whereas the green tea extract has no effect. Interestingly, Xu et al. [44] recently reported that sub-minimal inhibitory concentrations of EGCG suppress the gene expression of L-methionine-α-deamino-γ-mercaptomethane lyase, a *P. gingivalis* enzyme involved in methyl mercaptan production.

Data presented in this study, strongly suggest that green tea polyphenols deserve to be considered as promising compounds for the management of halitosis. Additional polyphenols were also reported to possess beneficial properties in regard to halitosis. A licorice extract as well as isoflavanones (licoricidin and licorisoflavan A) isolated from this fraction were found to inhibit the growth of *S. moorei*, *P. gingivalis*, and *Prevotella intermedia* as well as to dose-dependently reduce VSC production by these three bacterial species [45]. Moreover, Forrer et al. [46] recently reported that tea tree oil and alpha-bisabolol, plant essential oils contained in tongue gels and toothpastes, exert a bactericidal effect on *S. moorei* and therefore can be effective against halitosis. Further studies should evaluate whether combining the above natural compounds may result in synergistic effects.

## Conclusions

The initial therapy for halitosis consists in a mechanical scraping of the dorsum of the tongue to remove the biofilm also called tongue coating. An antimicrobial approach may also contribute to decrease tongue coating and reduce oral malodor. Our study brought evidence to support that green tea extract or EGCG possess a number of properties that may contribute to reduce *S. moorei*-related halitosis. Therefore, these natural compounds may be of interest to be used to supplement oral healthcare products.
